# Antimicrobial Resistance and Biofilms Underlying Catheter-Related Bloodstream Coinfection by *Enterobacter cloacae* Complex and *Candida parapsilosis*

**DOI:** 10.3390/antibiotics11091245

**Published:** 2022-09-14

**Authors:** Matúš Štefánek, Sigurd Wenner, Vítor Borges, Miguel Pinto, João Paulo Gomes, João Rodrigues, Isabel Faria, Maria Ana Pessanha, Filomena Martins, Raquel Sabino, Cristina Veríssimo, Isabel D. Nogueira, Patrícia Almeida Carvalho, Helena Bujdáková, Luisa Jordao

**Affiliations:** 1 Department of Microbiology and Virology, Faculty of Natural Sciences, Comenius University in Bratislava, 842 15 Bratislava, Slovakia; 2 SINTEF Industry, NO-7491 Trondheim, Norway; 3 Genomics and Bioinformatic Unit, Department of Infectious Diseases (DDI), National Institute of Health Dr. Ricardo Jorge (INSA), 1649-016 Lisbon, Portugal; 4 Unidade Laboratorial Integrada de Microbiologia, Department of Infectious Diseases (DDI), National Institute of Health Dr. Ricardo Jorge (INSA), 1649-016 Lisboa, Portugal; 5 Laboratório de Microbiologia e Biologia Molecular do Serviço de Patologia Clínica, Centro Hospitalar de lisboa Ocidental (CHLO), 1349-019 Lisboa, Portugal; 6 Direção do Programa de Prevenção e Controlo de Infeção e Resistência aos Antimicrobianos, Centro Hospitalar de lisboa Ocidental (CHLO), 1349-019 Lisboa, Portugal; 7 Reference Unit for Parasitic and Fungal Infections, Department of Infectious Diseases, National Institute of Health Dr. Ricardo Jorge (INSA), 1649-016 Lisboa, Portugal; 8 Institute of Environmental Health, Faculty of Medicine, University of Lisbon, 1649-028 Lisbon, Portugal; 9 MicroLab, Instituto Superior Técnico, 1649-001 Lisboa, Portugal; 10 Unidade de Investigação & Desenvolvimento, Departamento de Saúde Ambiental, Instituto Nacional de Saude Dr. Ricardo Jorge (INSA),1649-016 Lisboa, Portugal

**Keywords:** biofilm, catheter-related bloodstream infections, polymicrobial biofilms, antimicrobial resistance, microscopy, whole genome sequencing

## Abstract

Biofilm-associated infections are a public health concern especially in the context of healthcare-associated infections such as catheter-related bloodstream infections (CRBSIs). We evaluated the biofilm formation and antimicrobials resistance (AMR) of *Enterobacter cloacae* complex and *Candida parapsilosis* co-isolated from a CRBSI patient. Antimicrobial susceptibility of central venous catheters (CVCs) and hemoculture (HC) isolates was evaluated, including whole genome sequencing (WGS) resistome analysis and evaluation of gene expression to obtain insight into their AMR determinants. Crystal violet assay was used to assess dual biofilm biomass and microscopy was used to elucidate a microorganism’s distribution within biofilms assembled on different materials. Bacteria were multidrug-resistant including resistance to colistin and beta-lactams, likely linked to the mcr-9-like phosphoethanolamine transferase and to an ACT family cephalosporin-hydrolyzing class C beta-lactamase, respectively. The R398I and Y132F mutations in the *ERG11* gene and its differential expression might account for *C. parapsilosis* resistance to fluconazole. The phenotype of dual biofilms assembled on glass, polystyrene and polyurethane depends on the material and how biofilms were initiated by one or both pathogens. Biofilms assembled on polyurethane were denser and richer in the extracellular polymeric matrix, and microorganisms were differently distributed on the inner/outer surface of the CVC.

## 1. Introduction

Bloodstream infection (BSI) is a serious clinical condition that affected 8.3% of intensive care unit (ICU) patients in 2017 with 37% of the cases being catheter-related [1]. Although recent official data are missing, several studies suggest that during the COVID-19 pandemic the incidence of catheter-related bloodstream infections (CRBSIs) increased [2,3]. Most CRBSIs are monomicrobial with only one etiological agent identified. Coagulase-negative staphylococci are responsible for the majority of the cases (23.6%), but among the ten most frequent etiological agents are ESKAPE pathogens (*Enterococcus faecium*, *Staphylococcus aureus*, *Klebsiella pneumoniae*, *Acinetobacter baumannii*, *Enterobacter* spp. and *Pseudomonas aeruginosa*), *Escherichia coli*, *Serratia* spp. and *Candida* spp. [1]. Several studies reported the occurrence of polymicrobial CRBSI, as well as the increase in their incidence [4,5,6].

Medical devices, such as central venous catheters (CVCs), are often contaminated and colonized by biofilms that could originate biofilm-related infections [7]. Biofilms are microbial communities attached to a surface together with their secreted polymers. The development of these communities, namely polymicrobial communities, within the human body, particularly of immunocompromised individuals, could represent a huge threat. The levels of antimicrobial agent resistance (AMR) among the etiological agents previously referred in healthcare-associated infections (HAIs) remain high or very high [1,8], and biofilms are known to be a defense strategy against stress factors such as antimicrobial agents. Microorganisms within biofilms are known to be refractory to antimicrobials and host immune response [9].

In the present study, we aim at contributing to a better understanding of the role played by CVC colonization, namely by polymicrobial biofilms on CRBSI and AMR using the two etiological agents of a CRBSI case, namely *Enterobacter cloacae* complex and *Candida parapsilosis*. The multi-resistant and biofilm-forming *E. cloacae* complex isolates belong to the category of high-risk sources of nosocomial BSI [10,11]. The pathogenic potential of these bacteria increases their ability to spread resistance determinants through conjugative plasmids, not only within *E. cloacae* species, but also among other members of the *Enterobacteriaceae* family [12,13]. The yeast *C. parapsilosis* is the most common source of BSI among *Candida* spp. [14,15]. Moreover, it is often a strong biofilm producer known to colonize medical devices [16]. These two etiological agents of a CRBSI case (isolated from peripheral blood and CVC), were characterized using molecular analysis such as whole genome sequencing (WGS) and quantitative real-time PCR (qPCR) to evaluate their resistome and to establish whether the BSI was related to CVC colonization. In addition, biofilm assembly of the already mentioned pathogens was studied *in vitro*.

## 2. Results

### 2.1. CRBSI Aetiological Agents

The two aetiological agents, *E. cloacae* complex and *C. parapsilosis*, of a CRBSI defined as a BSI occurring 48 h before or after CVC removal, and with a positive culture of the same microorganism from either a CVC, or blood, or pus from the insertion site or differential delay positivity of blood samples [17] were studied. WGS of the microorganisms isolated from blood (hemoculture: HC) and CVC removed from the left subclavian vein after 4 days of dwell time was performed. Comparative genomics confirmed the isogenicity of the CVC/HC isolate pair (no fixed mutations were detected between the two genome sequences for both the *E. cloacae* complex and *C. parapsilosis*), thus supporting the scenario of CVC-mediated bloodstream bacterial/fungal coinfection. *C. parapsilosis* isolates were confirmed *in silico* to belong to group I-*C. parapsilosis sensu stricto* [18]. *E. cloacae* complex isolates belong to ST599 and were identified as *Enterobacter bugandensis*. Indeed, NCBI BLAST screening of large assembled contigs, as well as of the loci most commonly used for *E. cloacae* complex taxonomic assignment (*rpoB*, *gyrB*, *infB*, *atpD*, *hsp60* and *16SrRNA* [19,20]) had always rendered *Enterobacter bugandensis* as first hits, including the *E. bugandensis* EB-247 type strain [21] (% of nucleotide identity against this type strain: 99.58% for *rpoB*, 98.67% for *gyrB*, 99.70% for *infB*, 99.57% for *atpD*, 99.51% for *hsp60* and 99.86% for *16SrRNA*).

### 2.2. Antimicrobial Agent Resistance

The antimicrobial resistance profiles of the *E. cloacae* complex (*E. bugandensis*) (Table 1) and *C. parapsilosis* (Table 2) isolated from HC and CVC are shown. Clinically relevant AMRs were found for both etiological agents. The antimicrobial susceptibility profiles of *E. cloacae* complex (*E. bugandensis*) were similar, presenting resistance to at least five different antimicrobials including different beta-lactam antibiotics and the reserve antibiotic colistin. Of note, both isolates were found to be susceptible to the fourth generation cephalosporin (cefepime) and carbapenems (imipenem and meropenem). An *in silico* search for potential AMR genetic determinants allowed the detection of a beta-lactamase coding gene (locus_tag Eccomp_CVC_2017_38730) with 100% sequence identity and coverage to an ACT family cephalosporin-hydrolyzing class C beta-lactamase (GenBank accession no. NG_050712.1 and KJ949108.1) likely contributing to the observed resistance to beta-lactams (Table S2). In addition, the absence of carbapenemases and extended spectrum beta-lactamase (ESBL) such as bla-IMP, bla-VIM, CTX-M could account for the susceptibility to carbapenems [22,23,24]. Regarding colistin, the resistant phenotype could be most likely linked to the presence of an *mcr-9-*like Phosphoethanolamine transferase coding gene (locus_tag Eccomp_CVC_2017_02730) (Table S2). WGS revealed additional potential AMR determinants, such as multiple efflux pumps, and a fosfomycin resistance determinant (*fosA*) (Table S2), denoting the multidrug resistance character of this CRBSI-causing *E. cloacae* complex (*E. bugandensis*). Of note, the *E. cloacae* complex (*E. bugandensis*) CVC isolate was revealed to be resistant to the combination of fifth generation cephalosporin (ceftolozane) with the beta-lactamase inhibitor (tazobactam), when compared to HC-isolate, although the difference between the minimal inhibitory concentrations (MIC) was not high (2 mg/L versus 1 mg/L). In an attempt to find the genetic basis for this observation, we searched for non-fixed mutations and found a heterogenous SNV at nucleotide position 443 of the *rpoS* gene in the HC isolate (113,579 bp position in contig 11 of CVC isolate genome annotation). Importantly, this allelic admixture (G443A at ~60% frequency) leads to *rpoS* truncation at 148 aa position, which could explain the loss of resistance phenotype. Following this observation, the HC isolate was re-cultured to select multiple colonies for further AMR testing and WGS. Although different susceptibility profiles were observed across the 14 clones, all sequenced populations revealed a large deletion (position 79490-113510 bp in contig 11 of CVC isolate genome) also truncating *rpoS*. In summary, considering that WGS was performed over cultured isolates (selected after colony picking and short culture passaging), we hypothesize that the initially observed SNP-mediated *rpoS* inactivation, as well as the subsequent inactivating deletion event (and additional mutations observed across the HC colonies), may reflect *in vitro* adaptation rather than microevolution on the course of BSI that led to the loss of the resistance phenotype. Still, it is well documented that loss of *rpoS* as a response to environmental changes, either in natural or laboratory populations, affects the regulation of multiple genes, with potential impact on AMR [25,26], so our results warrant further investigation.

*C. parapsilosis* isolates share a common antimicrobial profile in which the high MIC to fluconazole (FLU > 256 mg/L) stands out. Azole resistance in *C. parapsilosis* is well documented, being at the molecular level associated with two multidrug transporters (Cdr1 and Mdr1) and an enzyme involved in ergosterol biosynthesis (lanosterol 14α-demethylase coded by the *ERG11* gene) [27,28,29].

For this reason, the expressions of genes coding for both efflux transporters, namely *CDR1* and *MDR1*, were screened. Moreover, a search for the presence/absence of point mutations in the *ERG11* gene was conducted. Both *C. parapsilosis* isolates were resistant to FLU (Table 2) and harbored the Y132F and R398I mutations due to the following nucleotide substitutions A395T and G1193T, respectively. This observation is in good agreement with other studies [29,30,31].

**Figure 1:**
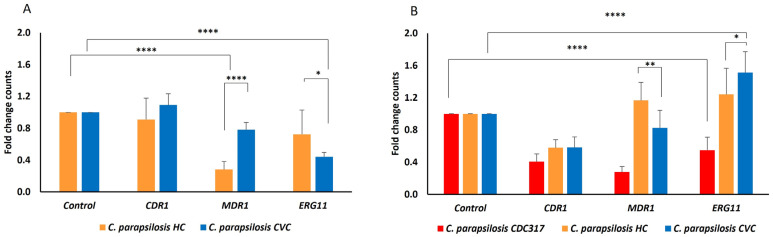
Relative fold-change in gene expression of *C. parapsilosis*. (**A**) Chart represents relative changes in expression of the *CDR1*, *MDR1* and *ERG11* genes in isolates of *C. parapsilosis* HC and CVC compared to standard *C. parapsilosis* CDC317 that was set up as a control and normalized to the value of 1. (**B**) Chart represents relative changes in expression of the *CDR1*, *MDR1* and *ERG11* genes in CDC317 strain, isolates of HC and CVC after 5 h incubation in the presence of sub-inhibitory concertation of FLU (2 µg/mL) compared to non-treated samples of corresponding isolates set to 1. A *p* < 0.05 (*) was considered statistically significant; *p* < 0.01 (**) highly significant; *p* < 0.0001 (****) highly extremely significant.

The expression of all three genes was also tested in the presence of a sub-inhibitory concentration of FLU. Results are summarized in Figure 1. The *MDR1* and *ERG11* gene expression was significantly downregulated in *C. parapsilosis* cultivated without FLU (Figure 1A) in comparison to the control. No significant difference was found for the *CDR1* gene expression. In the presence of a sub-inhibitory concentration of FLU (Figure 1B), regulation was slightly increased in the *MDR1* gene in *C. parapsilosis* HC (1.2-fold) and in the *ERG11* gene in both *C. parapsilosis*, HC and CVC (1.2 and 1.5-fold, respectively) compared to the same isolate without FLU.

### 2.3. Central Venous Catheter Colonization and CRBSI

**Figure 2:**
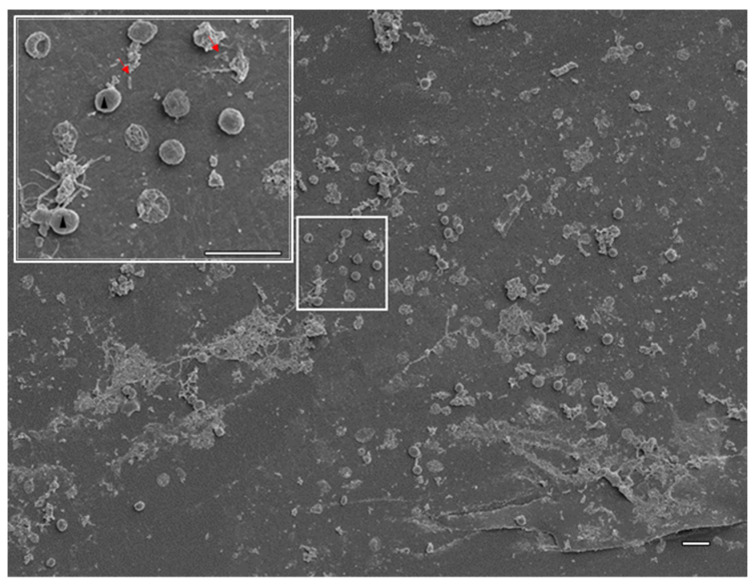
Central venous catheter colonization. Representative scanning electron micrograph of the lumen of a CVC removed from a patient with a catheter-related bloodstream infection. Rod-shaped bacteria (red arrows) and fungi (black arrow heads) colonized the CVC coexisting with host factors such as red blood cells, as can be observed with more detail in the inset. Scale bar: 10 µm.

Medical devices including CVCs are prone to microbial colonization that could evolve into biofilm and potentially biofilm-related infections [7]. Since biofilms are associated with increased AMR and multidrug-resistant microorganisms were isolated, we decided to screen the CVC for biofilm presence using scanning electron microscopy (SEM). The analysis revealed conditioning of CVCs’ lumen surface with host-derived factors (e.g., red blood cells, protein components) and that microbial colonization has begun (Figure 2). Based on morphology, *C. parapsilosis* cells were in the majority but rod-shaped bacteria, consistent with *E. cloacae* complex, were also present. The observed structures are not compatible with mature biofilm but could correspond to an early stage of biofilm formation when microorganisms adhere to the surface. This hypothesis led us to investigate *in vitro* the ability of these two microorganisms to assemble dual biofilms.

### 2.4. Dual Biofilms of E. cloacae Complex (E. bugandensis) and C. parapsilosis

**Figure 3:**
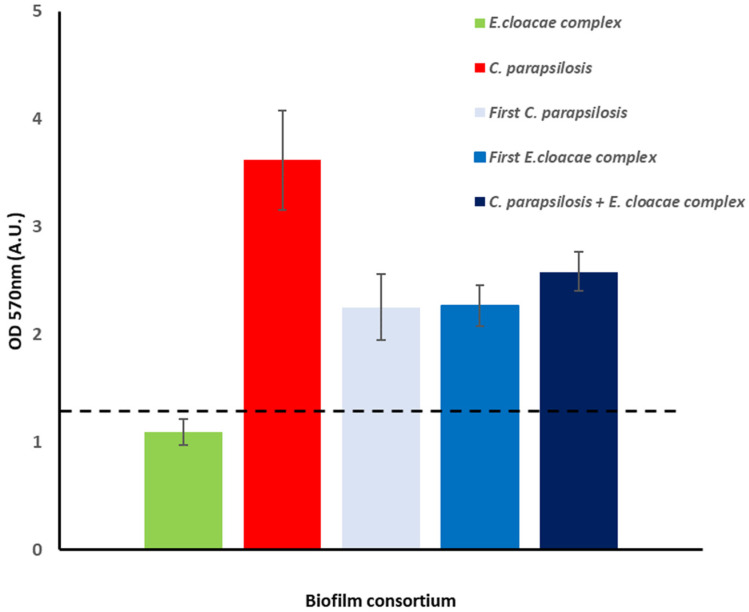
Biofilm assay. Single and dual biofilms of *E. cloacae* complex (*E. bugandensis*) and *C. parapsilosis* were monitored using crystal violet assay. Dual biofilms were either initiated by *C. parapsilosis* (First *C. parapsilosis*) or *E. cloacae* complex (First *E. cloacae* complex) or both microorganisms (*C. parapsilosis* + *E. cloacae* complex). The dashed line (---) highlights the OD_570nm_ value corresponding to the cut-off value for strong biofilm producers (SBP) according to Stepanović criteria [32]. A.U.: arbitrary units.

**Figure 4:**
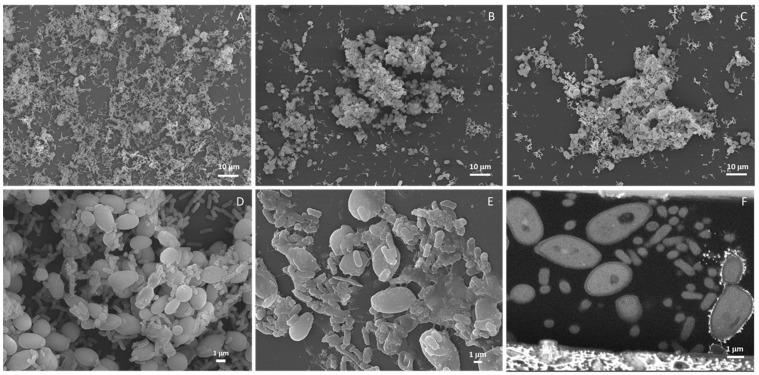
Dual biofilms. Biofilm started by a mix of *C. parapsilosis* and *E. cloacae* complex (*E. bugandensis*) (**A**), *C. parapsilosis* (**B**) or *E. cloacae* complex (*E. bugandensis*) (**C**) on polystyrene plates. Detail of mixed biofilm, started by the two microorganisms, on polystyrene (**D**) and polyurethane (**E**) acquired by SEM. Section of a dual biofilm assembled in the lumen of a polyurethane central venous catheter (**F**) acquired by FIB-SEM.

The ability of the *E. cloacae* complex (*E. bugandensis*) and *C. parapsilosis* isolated from HC to assemble biofilms on polystyrene (PS) was evaluated (Figure 3). Both microorganisms were able to assemble biofilms although, according to Stepanović‘s classification [32], *C. parapsilosis* (strong biofilm producer—SBP) has a better performance than *E. cloacae* complex (*E. bugandensis*) (moderate biofilm producer—MBP). Next, we evaluated the assembly of dual biofilms initialized by either the *E. cloacae* complex (*E. bugandensis*), *C. parapsilosis* or both microorganisms. In all tested conditions, the microbial consortium was classified as SBP having no significant differences in the biomass found with the two-tailed Student’s t-test. Nevertheless, under an optical microscope, few differences were noticed (data not shown). A more detailed analysis with SEM (Figure 4) confirmed our observation. When biofilms were started by both microorganisms, a more homogeneous colonization of the surface was observed (Figure 4A) than when biofilms were initialized by either *C. parapsilosis* (Figure 4B) or the *E. cloacae* complex (*E. bugandensis*) (Figure 4C). For further studies, namely distribution of microorganisms within the biofilm, we started biofilm assembly simultaneously with both pathogens. For the most studied *C. albicans*, it is known that biofilm formation is linked to the transition from yeast to hyphal mode [33]. In dual biofilms of *C. albicans* and bacteria, different distributions have been described, such as preferential adherence to hyphae for streptococci [34,35] and *S. aureus* [36], adherence to both hyphae and yeast forms for *E. coli* [5,37], *Clostridium perfringens*, *Bacteroides fragilis*, *Enterococcus faecalis* or *K. pneumoniae* [37]. Here, for dual biofilms of *C. parapsilosis*, generating only pseudohyphae, and *E. cloacae* complex (*E. bugandensis*), we observed that the bacterium adheres to yeasts, but a more detailed observation of biofilms suggested that the bacterium predominates on the bottom layer of the biofilms. This was observed for not only biofilms assembled on PS (Figure 4A–D) but also on polyurethane, the material the CVCs (Figure 4E) were made of. On this material, the presence of higher amounts of extracellular matrix was manifested. Biofilms assembled on the CVC were observed not only on the outer surface (Figure 4E), but also within the lumen (Figure 4F).

**Figure 5:**
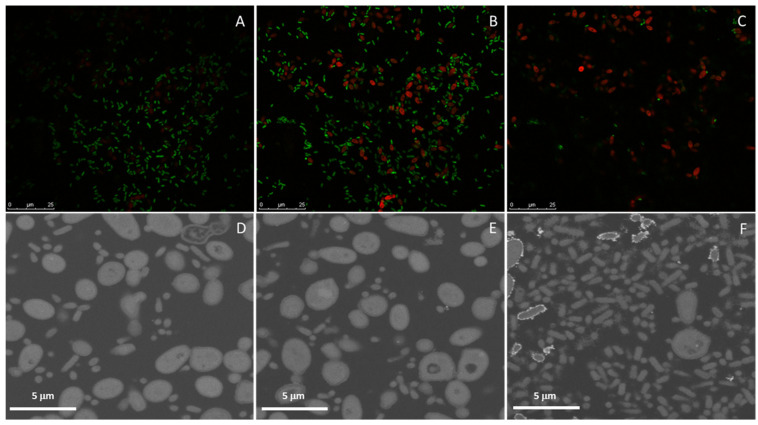
Dual biofilms of *E. cloacae* complex (*E. bugandensis*) and *C. parapsilosis* assembled on glass and polyurethane. Biofilms assembled on glass were monitored using FISH with the bottom (**A**), intermediate (**B**) and top layers (**C**) shown with *E. cloacae* complex (*E. bugandensis*) (green) and *C. parapsilosis* (red). Biofilms assembled within the polyurethane CVC lumen were monitored using FIB SEM with the bottom, intermediate and top layers shown in (**D**,**E**,**F**), respectively.

In order to better elucidate the distribution of both microorganisms within dual biofilms in different materials, two different methodologies were adopted. For glass, a light transparent material, biofilm assembly was monitored with fluorescence in situ hybridization (FISH) combined with scanning confocal microscopy whereas, for polyurethane, light opaque material, focused ion beam-scanning electron microscopy (FIB-SEM) was used. On the glass, preferential presence of the *E. cloacae* complex (*E. bugandensis*) on the biofilm’s bottom layer previously described for PS was observed (Figure 5). In the bottom layer (Figure 5A), the *E. cloacae* complex (*E. bugandensis*) dominates over *C. parapsilosis*, a balance is reached in the middle (Figure 5B) and the reverse of the bottom layer is observed on the top layer (Figure 5C). This feature was not observed for the biofilms assembled within the CVC lumen (Figure 5 and Supplementary Data: Video S1). Indeed, the reverse distribution was observed with *C. parapsilosis* predominating at the bottom (Figure 5D) and middle of the biofilm (Figure 5E) and the *E. cloacae* complex (*E. bugandensis*) at the top (Figure 5F).

### 2.5. Host Factors and Biofilm

**Figure 6:**
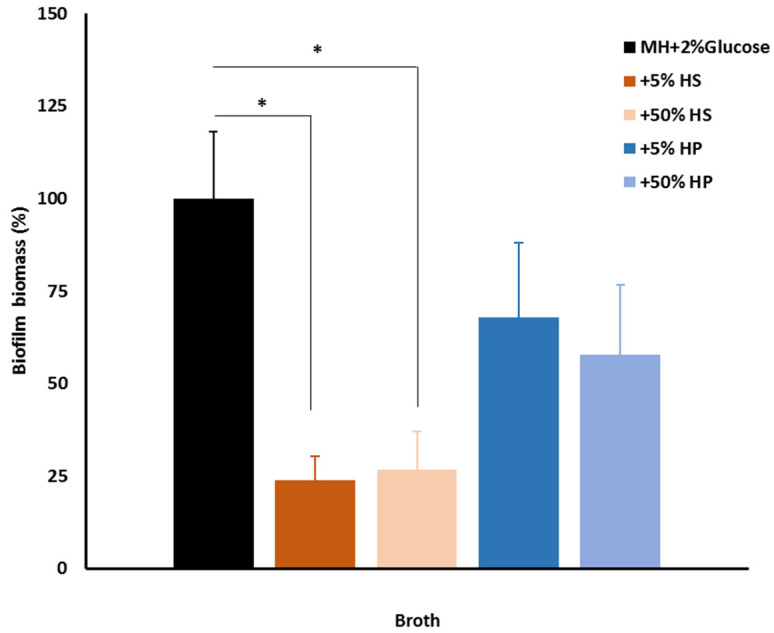
Impact of host factors on dual biofilm assembly. Dual biofilm biomass assessed with crystal violet assay on control (PS) and PS conditioned for 24 h with different concentrations of human serum (HS) and plasma (HP). A *p* < 0.05 (*) was considered statistically significant.

The different patterns of colonization observed on the CVC removed from the patient (Figure 2) and on different materials *in vitro* (Figure 4 and Figure 5) showed that surface properties play a role in the outcome. In the human body, the material will be exposed to host factors such as human serum and plasma that will condition the material surface, affecting its properties. Previously, Gominet and colleagues reported that after insertion CVC surface properties are immediately altered by the adhesion of host factors and as soon as 24 h after insertion CVCs could be already colonized by microorganisms [7]. For this reason, we decided to assess the effect of pre-conditioning the PS surface with different concentrations of human serum (HS) and human plasma (HP) on dual biofilm assembly started simultaneously by the *E. cloacae* complex (*E. bugandensis*) and *C. parapsilosis*. The obtained results showed that independently of the used concentration a significant reduction in biofilm biomass in comparison to the control (non-conditioned PS) was observed for HS but not for HP (Figure 6). This result shows that host factors play a role in the colonization of materials, and they will influence biofilm assembly on medical devices placed within the human body. In the present case, conditioning of the CVC lumen by a host factor could account for the differences in the colonization observed *in vivo* and *in vitro*. Nevertheless, we are aware that this is a complex and multifactorial process difficult to predict based on *in vitro* experiments.

## 3. Discussion

CRBSIs are a serious healthcare concern, especially among intensive care unit patients and patients with comorbidities such as renal impairment, cancer and diabetes [38,39]. Polymicrobial infections should represent a major challenge compared to monomicrobial infections although there is no conclusive evidence for this [40,41]. Another assumption often made is that most infections linked to medical devices are biofilm-related, but methods to diagnose these infections, and even to provide evidence that biofilms are assembled on the medical devices, are still scarce and urgently needed [42]. 

In the present work using SEM, we showed that only the inner surface of CVC was colonized by microorganisms (Figure 2). The presence of rod-shaped bacilli and yeast was consistent with the identified etiological agents: the *E. cloacae* complex (*E. bugandensis*) and *C. parapsilosis,* but the observed microbial communities were not consistent with mature biofilms. Attached microorganisms were predominantly *C. parapsilosis*, which is consistent with published reports on the enhanced ability of this microorganism to form biofilms on medical devices [38]. The use of WGS confirmed the identity between the microorganisms isolated from the CVC and HC, confirming the CVCs mediated BSI. The higher discriminatory ability of this technique over biochemical and matrix-assisted laser desorption ionization–time of flight (MALDI-TOF) identification techniques used in routine microbiology laboratories allowed the identification of, to the best of our knowledge, the first *E. bugandensis* isolate in Portugal. This species of nosocomial pathogen from the *Enterobacter cloacae* complex and its genome were reported for the first time in 2016 and 2019, respectively [43]. The fact that identification at the species level for the 22 spp. of the *E. cloacae* complex is not achieved with routine techniques highlights the importance of gradually introducing, and balancing the cost/benefit of introducing, WGS in clinic diagnosis. Resistome analysis allowed confirmation of the AMR phenotypes observed for this bacterium. Here, we highlight the fact that both isolates were resistant to the reserve antibiotic colistin, which can represent a concerning challenge for infection control. An additional difficulty was brought by *C. parapsilosis* isolates’ resistance to FLU, mediated by Y132F and R398I mutations in the *ERG11* gene. To the best of our knowledge, this is the first report in Portugal of the detection of these two mutations linked to azole resistance, although previously described for *C. parapsilosis* responsible for BSI in other countries [30,31]. Moreover, a recent study monitoring the antifungal resistance in *C. parapsilosis* isolates at Madrid metropolitan area hospitals for the last three years proved that isolates used for sequencing the *ERG11* gene mainly harbored the Y132F Erg11p amino acid substitution [29]. Results obtained from qPCR analysis of both *C. parapsilosis* isolates suggested that regulation of tested genes does not play an important role in FLU resistance, and probably, found mutations in the *ERG11* gene (Y132F and R398I) were responsible for resistance to FLU. 

A coinfection by two AMR pathogens is a challenge that could be worsened by their persistence within a biofilm structure known to favor horizontal gene transfer and protect microorganisms from antimicrobials and the host immune system. For this reason, we decided to study dual biofilm assembly *in vitro* on different surfaces. Although it has been shown that biofilm assembly *in vitro* is a poor predictor of biofilm assembly *in vivo* for several microorganisms [44] and for catheter-related candidemia [45], it is a valuable tool to start elucidating the interaction between the different microorganisms. On the model surface PS, both microorganisms were able to assemble biofilms although *C. parapsilosis* had a better performance (Figure 3) in good agreement with the previous reports of its enhanced ability to assemble biofilms on different surfaces [46,47]. The dual biofilm biomass was independent of the biofilm being started by either the bacterium or yeast alone or together (Figure 3) but the observed biofilm phenotypes suggested otherwise (Figure 4). More homogenous colonization of the PS surface was achieved with biofilms started simultaneously by both microorganisms and the *E. cloacae* complex (*E. bugandensis*) appears more often attached to the surface (Figure 4A,D). If we take as model polymicrobial oral biofilms, the *E. cloacae* complex (*E. bugandensis*) might be an early colonizer and *C. parapsilosis* might be a late colonizer [48]. Considering that the bacterium is a moderate and the yeast a strong biofilm assembler (Figure 3), this hypothesis looks surprising. Nevertheless, this microbial distribution within biofilms was observed not only on PS but also on glass (Figure 5A–C) and polyurethane (Figure 4E). On polyurethane, a difference was noticed: the exacerbated presence of the extracellular matrix. Since the three materials have similar properties in terms of surface charge and hydrophobicity, the differences in shape might explain the observed result [49]. Indeed, both PS and glass were flat, whereas polystyrene has a tubular shape (CVC segment). The role played by shape might also contribute to explaining the differences in biofilm occurrence in the CVC lumen and outer surface. As mentioned before, only the CVC lumen was colonized *in vivo* (Figure 2) and *in vitro* microorganisms’ distribution within the biofilm was different on both surfaces. On the outer and inner surfaces, the bacterium and the yeast were predominantly found in the biofilm’s bottom layer, respectively. The shape could play a key role but for the *in vivo* result, the conditioning of the material surface by a host factor must be considered. The influence of the host is crucial and very difficult to mimic *in vitro* since each host is unique. Although being limiting, we decided to evaluate the effect of conditioning the polyurethane surface with serum and plasma from healthy donors. The observed results (Figure 6) showed that PS conditioning by serum and plasma by itself has an impact on biofilm assembly. More than an explanation, these results show the limitation of our model that does not evaluate the role played by flow, host microbiome and other factors on CVC colonization. It also supports the importance of developing new materials as a strategy to mitigate CRBSI [50], shows the need to evaluate the impact of using CVCs’ locking solutions on CVC surface and shows the need to develop in vitro models that could mimic more closely the *in vivo* situation.

## 4. Materials and Methods

### 4.1. Microorganism Isolation and Culture

Four clinical isolates, two *Candida parapsilosis* and two *Enterobacter cloacae* complexes, with one of each being isolated from peripheral blood and central venous catheter (CVC—4 days dwell time), recovered from a patient with a CRBSI, were used in this study. Bact/Alert system (bioMérieux, Marcy l’Etoile, France) and the Maki reference method on blood agar [51] were used to culture peripheral blood and CVC, respectively. ViteK2 or ViteK-MS (bioMérieux, Marcy l’Etoile, France) were used to identify microorganisms following the manufactures’ instructions. Mueller Hinton (MH) broth or agar and Sabouraud with chloramphenicol (Sab-Chl) broth or agar were used to grow *E. cloacae* complex and *C. parapsilosis*, respectively. All microorganisms were preserved at −20 °C in Tryptic Soy broth (TSB) with 20% glycerol. *C. parapsilosis* CDC317 standard strain (kindly provided by Prof. J. Nosek, DrSc, Department of Biochemistry, Faculty of Natural Sciences, Comenius University in Bratislava, Slovakia) was used as a control strain in experiments conducted to elucidate FLU resistance mechanism.

### 4.2. Antimicrobial Susceptibility Tests

The antimicrobial susceptibility activity of *E. cloacae* complex (2 isolates) was assessed by the minimum inhibitory concentration (MIC) determination system (Vitek 2 system). Briefly, an inoculum of 1 × 10^5^ bacteria/mL and AST-N203 and AST-N273 cards (BioMerieux, Marcy l’Etoile, France) were used, with the results being interpreted according to EUCAST guidelines [52]. Susceptibility to colistin was determined and interpreted according to EUCAST guidelines [52] for the clinical strains of *E. cloacae*. Briefly, broth microdilution method used MH broth, a concentration range between 0.155 and 8 µg/mL, an inoculum of 5 × 10^5^ bacteria/mL incubated overnight (ON) at 37 °C and the susceptible *E. coli* ATCC 25922 as a control.

The antimicrobial susceptibility activity of *C. parapsilosis* was assessed with MIC determination using E-test strips (BioMerieux) according to the manufacturer’s instructions. Briefly, a 0.5 McFarland suspension was inoculated onto RPMI agar supplemented with 2% glucose (BioMerieux); incubation was performed at 37 °C for 24–48 h. Results were interpreted according to EUCAST guidelines [53].

### 4.3. Biofilm Assay

Dual biofilm was prepared for 48 h according to [33,54] with modifications. *C. parapsilosis* and *E. cloacae* complex isolates from hemoculture (HC) were grown ON in Sab-Chl or MH broth, respectively, at 37 °C with aeration. Pellets of *C. parapsilosis* and *E. cloacae* complex were obtained by centrifugation at 3000× *g* for 5 min or 800× *g* for 10 min, respectively, and washed twice with phosphate buffer saline (PBS). Then, *C. parapsilosis* cells were enumerated using a Neubauer modified chamber and a 2 × 10^6^ cells/mL suspension was prepared by dilution in MH broth supplemented with 2% glucose (MH- 2% glucose). For *E. cloacae*, a bacterial suspension with an OD_600 nm_ of 0.4 was prepared in MH broth supplemented with 2% glucose (MH-2% glucose). For biomass determination, crystal violet assay was performed in 96-well polystyrene plates. For single microorganism biofilms, either 200 µL suspension of *C. parapsilosis* or *E. cloacae* complex were added to the wells and allowed to adhere for 2 h at 37 °C. After the adherence phase, the media were removed, non-adherent microorganisms were removed by washing with PBS, new pre-warmed MH-2% glucose was added, and the biofilm was incubated further until 48 h had elapsed from the beginning of the experiment. For dual biofilms, we adopted two protocols: (i) starting with 100 µL suspension of each microorganism, followed by the procedure described for single microorganism biofilms or (ii) starting with 200 µL of one of the microorganisms, incubation for 2 h at 37 °C, remotion of non-adherent microorganisms by washing with PBS, allowed the biofilm to form for 24 h before adding the second microorganism and repetition of the procedure (2 h adherence, washing, incubation at 48 h). At 48 h, biofilms were washed thrice with PBS, stained for 15 min with 100 µL of 1.4% crystal violet, washed twice with PBS with the dye being allowed to dissolve in 95% ethanol for 15 min and titrated at OD_570nm_, as previously described [55]. Results were interpreted according to Stepanović and coauthors [32]. Briefly, the cut-off value (ODc) was defined as three standard deviations (SDs) above the mean OD of the blank. Based upon the previously calculated OD values (ODs) for different conditions, results were interpreted as follows: ODs ≤ ODc non-biofilm producer (NBP); ODc < ODs ≤ 2ODc weak biofilm producer (WBP); 2 ODc < ODs ≤ 4 ODc moderate biofilm producer (MBP); 4 ODc < ODs strong biofilm producer (SBP). At least three independent experiments in triplicate were conducted.

### 4.4. Biofilms and Host Factors

The effect of inactivated human plasma (HP) and heat-inactivated human AB serum (HS) on the assembly of dual biofilms started with *E. cloacae* complex and *C. parapsilosis* was evaluated. HP with a fibrinogen concentration of 2.59 g/L donated by Hemovida^®^ and HS was purchased from Sigma-Aldrich (Burlington, NJ, USA). Ninety-six-well polystyrene (PS) plates were conditioned with 5 or 50% solutions of HP or HS in MH- 2% glucose for 2 h at 37 °C. Then, dual biofilms were assembled as described under biofilm assay using non-conditioned wells as control and MH-2% glucose as media. After 48 h, biofilm assembly was monitored with crystal violet assay. The effect of HP and HS on biofilm assembly was assessed by calculating the percentage of biofilm biomass assembled on each condition compared to the control (biofilm assembled on unconditioned PS). 

### 4.5. Analysis of Biofilm Assembled In Vitro on Different Surfaces

PS (plates) or glass coverslips and tips of polyurethane CVCs (approximately 1 cm long) were deposited on the wells of 24-well plates with the protocol for biofilm assembly being performed as previously described adjusted to the working volume of 1 mL. Biofilms were then processed for:

(i) Scanning electron microscopy (SEM). Samples were washed with PBS and fixed with 4% paraformaldehyde (Merck, Darmstadt, Germany) in PBS for 30 min at room temperature (RT) protected from light. The fixative was removed, and the samples were washed twice in PBS for 10 min and post-fixed with 1% osmium tetroxide (EMS, Hatfield, PA, USA) in the same buffer for 90 min on ice protected from the light. Then, the samples were washed twice for 10 min at RT with PBS and twice with deionized water. Dehydration was performed at RT using serial dilutions of ethanol as follows: once in 50%, 70%, 80% and 95% ethanol for 30 min and twice in 100% ethanol for 30 min each. Samples were then trimmed (CVC was cut longitudinally), allowed to dry at RT, mounted on top of double-sided carbon tape (CVC was mounted in such a way that both inner and outer surfaces were visible), coated with 20 nm thick gold-palladium film using a sputter coater QISOT ES (Quorum Technologies, Laughton, UK) and analyzed under a scanning electron microscope, JSM-7100F (JEOL, Tokyo, Japan) using secondary electron detector.
(ii) Focused ion beam scanning electron microscopy (FIB-SEM) tomography. Samples were prepared as described previously for SEM until the second incubation with 100% ethanol. Then, Epoxy 812 resin (EMS) was added with the samples left uncapped overnight in a chemical chamber. The resin was then replaced by a new batch and further incubated for 3 h. Then, the sample and one drop of resin were compressed between 2 sheets of ACLAR film (EMS) allowing the sample to be encased in a thin resin layer. Samples were allowed to polymerize at 65 °C. FIB-SEM tomography was performed with an FEI HELIOS G4 instrument (Thermo Fisher Scientific, Waltham, MA, USA). Slicing was performed with a Ga+ ion beam accelerated at 30 kV and a current of 1 nA, while imaging was performed with an electron beam accelerated at 5 kV, a current of 1.6 nA and using the backscattered electron detector. A slice thickness of 30 nm was used. The acquisition operation was controlled through the Auto Slice & View 4.0 software package and reconstruction was carried out with Avizo Fire software package. Individual fungi and bacteria were manually labelled for intensity thresholding and 3D volume reconstruction.
(iii) Fluorescence in situ hybridization (FISH)/laser scanning confocal microscopy. For biofilms assembled on glass coverslips, FISH was used to assess the distribution of fungi (*C. parapsilosis*-probe PF2) and bacteria (*E. cloacae* complex-probe EUB) using 5‘-labeled oligonucleotide probe fluorochromes FITC and Cy3, respectively (Biomers.net, Ulm, Germany). Non-sense probes labelled with the same fluorochromes were used as control. The probe sequences were previously described [56,57]. Samples were fixed as described for SEM, washed with PBS, permeabilized with 200 U/mL of lyticase (Sigma-Aldrich) followed by 10 mg/mL of lysozyme in hybridization buffer pH 8 (20 mM Tris-HCl pH8, 0.9 M sodium chloride, 20% formamide, 0.01% sodium dodecyl sulfate all from Sigma-Aldrich) for 15 min at 30 °C and incubated in a humidified chamber with 1 µM PF2 probe in the hybridization buffer for 3 h at 45 °C. After the first hybridization step, the unbound probe was washed off with a 45 °C pre-warmed washing buffer (20 mM Tris-HCl pH8, 0.212 M sodium chloride, 5 mM EDTA, 0.01% SDS) and water for 10 min each. Hybridization with 1 µM with EUB probe was performed for 2 h at 45 °C; the unbound probe was removed as previously. Coverslips were mounted by inversion on a drop of fluorescence-mounting medium previously placed on a glass slide. Samples were stored at 4 °C, protected from light, until visualization through a confocal microscope (Leica, SP2, Wetzlar, Germany) under an immersion objective of ×63 and an ocular objective of ×10.

### 4.6. Analysis of CVC Colonization

The CVC was washed with sterile saline solution and preserved in 4% paraformaldehyde (Merck, Darmstadt, Germany) in PBS at 4 °C protected from light until further processing. Then, the sample was processed as described previously for SEM in the Analysis of biofilm assembled *in vitro* on different surfaces, see Section 4.5.

### 4.7. DNA Extraction and WGS

Three milliliters of ON *C. parapsilosis* cultures (37 °C with aeration in Sab-chl broth) were harvested by centrifugation (10 min, 5000× *g*), re-suspended in 600 µL of lysis buffer (1 M sorbitol, 100 mM EDTA, 14 mM β-mercaptoethanol, 200 U of lyticase, all from Sigma, St. Louis, MO, USA) and incubated at 30 °C for 30 min. The sample was then centrifuged (10 min, 5000× *g*) with the pellet being processed according to the manufacturer’s instructions for the Qiagen DNeasy Blood & Tissue kit (Qiagen, Hilden, Germany). For *E. cloacae* complex isolates, the same DNA extraction kit was used starting with one loop of ON cultures on MH agar. DNA was subjected to Nextera XT library preparation (Illumina, San Diego, CA, USA) prior to paired-end sequencing (2 × 250 bp or 2 × 150 bp) on either a MiSeq or a NextSeq 550 instrument (Illumina, San Diego, CA, USA), according to the manufacturer’s instructions.

### 4.8. Genome Characterization

Genome sequences were assembled using INNUca v4.2.0 pipeline (https://github.com/B-UMMI/INNUca, accessed on 18 August 2022) [58] (assembly statistics are reported in Supplementary Table S1). In order to compare the genome background of the same-patient pairs of isolates (CVC versus HC), HC quality-processed reads were mapped against the polished genome assembly obtained from the respective CVC isolate using Snippy v. 4.5.1 (https://github.com/tseemann/snippy (accessed on 18 August 2022); --mincov 10 --minfrac 0.51 --mapqual 20 --basequal 20). The same procedure was also applied to inspect the rpoS mutational profile of multiple colonies of the cultured *E. cloacae* complex HC isolate, with mapping visualization being performed using Integrative Genomics Viewer (https://igv.org/app/, accessed on 18 August 2022). For *C. parapsilosis*, group confirmation was performed in silico using SnapGeneViewer v4.1 based on the BanI restriction enzyme digestion sites of gene SADH (CPAR2_800970), as previously described [18]. Regarding in silico AMR screening, for *C. parapsilosis* quality-processed reads were mapped against the reference genome of the *C. parapsilosis* CDC317 (GenBank accession numbers HE605202-HE605210) to check for the presence of AMR-related mutations described in the literature [47,59,60,61]. For *E. cloacae* complex, the polished genome was annotated using Prokka v1.14.5 [62] and MLST prediction was determined using mlst v2.16.1 software (https://github.com/tseemann/mlst, accessed on 18 August 2022). Species identification was performed with BLASTn-screening large contigs and loci commonly used for *E. cloacae* complex taxonomic assignment [19,20], namely: rpoB (locus_tag Eccomp_CVC_2017_44980), gyrB (locus_tag Eccomp_CVC_2017_26180), infB (locus_tag Eccomp_CVC_2017_10780), atpD (locus_tag Eccomp_CVC_2017_42980) and hsp60 (locus_tag Eccomp_CVC_2017_38850 and 16SrRNA. The polished assemblies were also queried against the Virulence Factor Database (VFDB) [63] and the several databases of AMR-associated markers (ResFinder, NCBI, ARG-ANNOT and CARD) using the BLASTn-based ABRIcate v0.9.8 tool (https://github.com/tseemann/abricate, accessed on 18 August 2022). Results of this screening, including the “locus_tags” of the targeted genes in the reference genomes and in the Prokka-annotated polished genome are presented in Supplementary Table S2. The *E. cloacae* complex assembled contigs (.fasta and .gbk files), the nucleotide sequences of the prediction transcripts (.ffn files) and the respective amino acid sequences of the translated CDS sequences (.faa files) of the CVC isolate are available at https://doi.org/10.5281/zenodo.6977339 (accessed on 18 August 2022). All sequencing reads generated in this study were deposited in the European Nucleotide Archive under BioProject accession no. PRJEB45360 (Table S1).

### 4.9. Isolation of RNA from C. parapsilosis

The two clinical isolates and the reference *C. parapsilosis* CDC317 strain were grown up to 16 h in 20 mL of YPD broth (1% yeast extract, Biolife, Italy; 2% mycological peptone, Lab M Limited, Buri, UK; 2% D-glucose, Centralchem, Bratislava, Slovakia) with/without subinhibitory concentration of FLU (2 mg/mL; Pfizer, New York, NY, USA) in an orbital shaker (Multitrone Standard, Infors HT, Bottmingen-Basel, Switzerland), at 180 rpm/30 °C. Then, density was adjusted to OD_560_ 0.02 and *C. parapsilosis* was further cultivated in YPD broth supplemented with 2% glucose with/without FLU (2 µg/mL) to reach the exponential phase (about 5 h). Then, the cultures were transferred to a new sterile 50 mL Falcon tube (Sarstedt AG & Co. KG, Nümbrecht, Germany) and centrifuged at 3000× *g*, 3 min at 15 °C (Universal 32R, Andreas Hettich GmbH & Co. KG, Tuttlingen, Germany). The supernatant was discarded, and cells were washed twice with PBS (MP Biomedicals, LLC, Irvine, CA, USA). After discarding supernatants, 1 mL of PBS was added to resuspend the cells. Then, the yeasts were transferred to new microcentrifuge tubes and centrifuged at 8000× *g*, 2 min, 15 °C. Afterwards, the supernatants were discarded, and the isolation of RNA was proceeded with the GeneJET RNA Purification Kit (Thermo Scientific, Waltham MA, USA) with the following modification: 200 µL of yeast lysis was added to resuspend the pellet followed by a 60 min incubation at 30 °C. All further steps were conducted by following the manufacturer’s protocol. Eluted RNA was then purified with DNase I (Thermo Scientific, Waltham, MA, USA) and samples were stored at −80 °C or used immediately in a downstream application. To obtain cDNA for qPCR experiments, a cDNA synthesis kit was used (Maxima First Strand cDNA Synthesis Kit, Thermo Scientific, Waltham, MA, USA) according to the manufacturer’s instructions. Synthesized cDNA was stored at −20 °C or used immediately in qPCR.

### 4.10. Evaluation of Gene Expression Related to FLU Resistance in C. parapsilosis

All used primers were synthesized according to previously published sequences [64], and the *ACT1* housekeeping gene was used as the control (all primers were synthesized by Metabion International AG, Planegg/Steinkirchen, Germany). For initial confirmation with PCR, a thermal protocol was set up (initial denaturation at 95 °C for 1 min followed by 40 cycles of denaturation at 90 °C for 15 s, annealing at 54 °C for 1 min and extension at 72 °C for 1 min; followed by a final extension at 72 °C for 10 min). Gel electrophoresis was performed to confirm product lengths (2% agarose gel, 120 V, 90 min). Afterwards, we set up a thermal protocol for 2-step qPCR (40 cycles of denaturation at 95 °C for 15 s and annealing at 54 °C for 1 min). For the reaction, HOT FIREPol^®^ EvaGreen^®^ qPCR Mix Plus (Solis BioDyne OÜ, Tartu, Estonia) and Mx3000P qPCR system (Agilent Technologies, Inc., Santa Clara CA, USA) was used. All data were analyzed by MxPro software provided by Agilent Technologies. Relative gene expression was calculated using the 2ΔΔCq method. *C. parapsilosis* CDC317 was set up as the control sample and normalized to a value of 1 (Figure 1A) and relative changes in expression of the *CDR1*, *MDR1* and *ERG11* genes in CDC317 strain, isolates HC and CVC after 5 h incubation in the presence of sub-inhibitory concertation of FLU (2 µg/mL) were compared to non-treated samples of corresponding isolates set to 1 (Figure 1B). All experiments were performed in three parallel wells and in six independent replicas.

### 4.11. Statistical Analysis

Results of at least three independent experiments of biofilm assembly were expressed as the means +/− standard deviation. Statistical significance was assessed by the Student t-test two-tailed. The statistical significance of qPCR data was assessed with Two-Way ANOVA using GraphPad Prism software (Graph Pad, San Diego CA, USA). A *p* < 0.05 (*) was considered statistically significant; *p* < 0.01 (**) highly significant; *p* < 0.001 (***) extremely significant; *p* < 0.0001 (****) highly extremely significant.

## 5. Conclusions

WGS could be an important tool for the identification of etiological agents of CRBSI (and other infections). This methodology allowed the linkage between the CVC colonizers and BSI, as well as insights into the genetic determinants of AMR. CVC colonization by microorganisms might contribute to CRBSIs, even when mature biofilms are not present. The phenotypes of dual biofilms assembled by the *E. cloacae* complex (*E. bugandensis*) and *C. parapsilosis* differ depending on how they are initiated, the presence of host factors, material properties and shape, highlighting the need for developing *in vitro* multifactorial models.

## Figures and Tables

**Table 1:** Antimicrobial susceptibility profiles of *E. cloacae* complex (*E. bugandensis*) isolates.

Source	Central Venous Catheter		Hemoculture	
Antibiotic	MIC (mg/L)	Phenotype ^1^	MIC (mg/L)	Phenotype
Ticarcillin	≥128	R	≥128	R
Piperacillin/Tazobactam	≥128	R	≥128	R
Ceftazidime	≥64	R	≥64	R
Ceftolozane/Tazobactam	2	R	1	S
Cefepime	0.25	S	≤0.12	S
Aztreonam	16	R	16	R
Imipenem	≤0.25	S	≤0.25	S
Meropenem	≤0.25	S	≤0.25	S
Amikacin	2	S	≤1	S
Gentamicin	≤1	S	≤1	S
Tobramycin	≤1	S	≤1	S
Ciprofloxacin	≤0.06	S	≤0.06	S
Levofloxacin	≤0.12	S	≤0.12	S
Tigecyclin	≤0.5	S	≤0.5	S
Trimethoprim/Sulfamethoxazole	≤20	S	≤20	S
Colistin	>8	R	>8	R

^1^ R: resistant; S: susceptible.

**Table 2:** *Candida parapsilosis* isolate susceptibility to antimicrobials.

Source	Central Venous Catheter		Hemoculture	
Antimicrobial Agents	MIC (mg/L)	Phenotype ^1^	MIC (mg/L)	Phenotype
Fluconazole	>256	R	>256	R
Amphotericin B	0.032	S	0.125	S
Anidulafungin	0.50	S	0.50	S

^1^ R: resistant; S: susceptible.

## Data Availability

All sequencing reads generated in this study were deposited in the European Nucleotide Archive under BioProject accession no. PRJEB45360.

## Supplementary Materials

The following supporting information can be downloaded at: https://www.mdpi.com/article/10.3390/antibiotics11091245/s1, Table S1: Isolate metadata, WGS, statistics and antimicrobial resistance phenotype/genotype data for *E. cloacae* complex (*E. bugandensis*) and *C. parapsilosis* isolates evaluated in this study; Table S2: List of genes likely associated with antibiotic resistance or virulence predicted in silico by querying the predicted CDSs (i.e, prokka “.ffn” files) of the *E. cloacae* complex (*E. bugandensis*) (ST599) strain evaluated in this study against the ResFinder, NCBI, ARGANNOT, CARD and VFDB databases. The table includes CDSs (“locus_tags” and product description) yielding a hit in at least one database (CDSs with more than one hit are highlighted in blue). Matches with <40% of query coverage and/or <40% of identity as well as Hits (AMR/Virulence gene) with matches for more than one CDS (within a given database) are highlighted in gray to denote that the “matching” result should be interpreted with caution. Due to the “isogenicity” of the CVC/HC isolates, annotation is only provided for the CVC isolate; Video S1: Tomogram of dual biofilm assembled in the CVC’s lumen acquired by FIB-SEM.
